# The identification of nuclear αvβ3 integrin in ovarian cancer: non-paradigmal localization with cancer promoting actions

**DOI:** 10.1038/s41389-020-00254-2

**Published:** 2020-07-29

**Authors:** Chen Seraya-Bareket, Avivit Weisz, Elena Shinderman-Maman, Sharon Teper-Roth, Dina Stamler, Nissim Arbib, Yfat Kadan, Ami Fishman, Debora Kidron, Evgeny Edelstein, Martin Ellis, Osnat Ashur-Fabian

**Affiliations:** 1 Translational Oncology Laboratory, Hematology Institute and Blood Bank, Meir Medical Center, 44821 Kfar-Saba, Israel; 2 Department of Human Molecular Genetics and Biochemistry, Sackler Faculty of Medicine, Tel Aviv University, 6997801 Tel Aviv, Israel; 3 Sackler Faculty of Medicine, Tel Aviv University, Tel Aviv, Israel; 4 Gynecologic Oncology Division, Department of Obstetrics and Gynecology, Meir Medical Center, 44821 Kfar Saba, Israel; 5 Department of Pathology, Meir Medical Center, 44821 Kfar Saba, Israel

**Keywords:** Ovarian cancer, Cell growth, Membrane trafficking

## Abstract

Nuclear translocation of transmembrane proteins was reported in high-grade serous ovarian cancer (HGSOC), a highly aggressive gynecological malignancy. Although the membrane receptor αvβ3 integrin is amply expressed in HGSOC and involved in disease progression, its nuclear localization was never demonstrated. Nuclear αvβ3 was explored in HGSOC cells (OVCAR3, KURAMOCHI, and JHOS4), nuclear localization signal (NLS) modified β3 OVCAR3, Chinese hamster ovaries (CHO-K1) and human embryonic kidney (HEK293) before/after transfections with β3/β1 integrins. We used the ImageStream technology, Western blots (WB), co immunoprecipitations (Co-IP), confocal immunofluorescence (IF) microscopy, flow cytometry for cell counts and cell cycle, wound healing assays and proteomics analyses. Fresh/archived tumor tissues were collected from nine HGSOC patients and normal ovarian and fallopian tube (FT) tissues from eight nononcological patients and assessed for nuclear αvβ3 by WB, confocal IF microscopy and immunohistochemistry (IHC). We identified nuclear αvβ3 in HGSOC cells and tissues, but not in normal ovaries and FTs. The nuclear integrin was Tyr 759 phosphorylated and functionally active. Nuclear αvβ3 enriched OVCAR3 cells demonstrated induced proliferation and oncogenic signaling, intact colony formation ability and inhibited migration. Proteomics analyses revealed a network of nuclear αvβ3-bound proteins, many of which with key cancer-relevant activities. Identification of atypical nuclear localization of the αvβ3 integrin in HGSOC challenges the prevalent conception that the setting in which this receptor exerts its pleiotropic actions is exclusively at the cell membrane. This discovery proposes αvβ3 moonlighting functions and may improve our understanding of the molecular basis of ovarian cancer pathogenesis.

## Introduction

High-grade serous ovarian cancer (HGSOC) is a gynecological malignancy that, although about one-tenth as common as breast cancer, is associated with a disproportionately large number of deaths due to late diagnosis after the disease has metastasized to distant sites^1^. Recent studies strongly suggest that the fallopian tube (FT) epithelium is the source of HGSOC, rather than the ovarian surface epithelium as previously believed^2^. The first step is a mutation in FT cells in the tumor suppressor gene p53, known as “p53 signature”, later additional mutations accumulate and the cells implant on the ovary, resulting in HGSOC tumor.

Emerging evidence has shown the translocation of transmembrane proteins from the cell membrane to the nucleus^3^. Such mislocalization of proteins has been suggested to alter the original protein activity and to be associated with several human diseases, including cancer^4^. Data on nuclear translocations of membrane receptors in HGSOC is limited to epidermal growth factor receptor (EGFR) and E cadherin^5,6^ and identification of additional nuclear receptor trafficking may lead to better understanding of disease pathogenesis. HGSOC metastasis is characterized by a spread of tumor cells inside the peritoneal cavity and their subsequent invasion within the organs of the pelvis^1^. These adhesion-related events are partly dependent on integrins, plasma membrane receptors with profound effects on cancer development^7^. Among the 24 integrins, αvβ3 integrin is amply expressed on the membrane of HGSOC cells and participates in disease progression and invasion^8–11^. We describe here the first indication for an intranuclear localization of the αvβ3 integrin, its interactions with an array of cancer-relevant proteins and a suggested role in HGSOC cell proliferation.

## Results

### αvβ3 integrin localizes in the nucleus of HGSOC cells

**Fig. 1:**
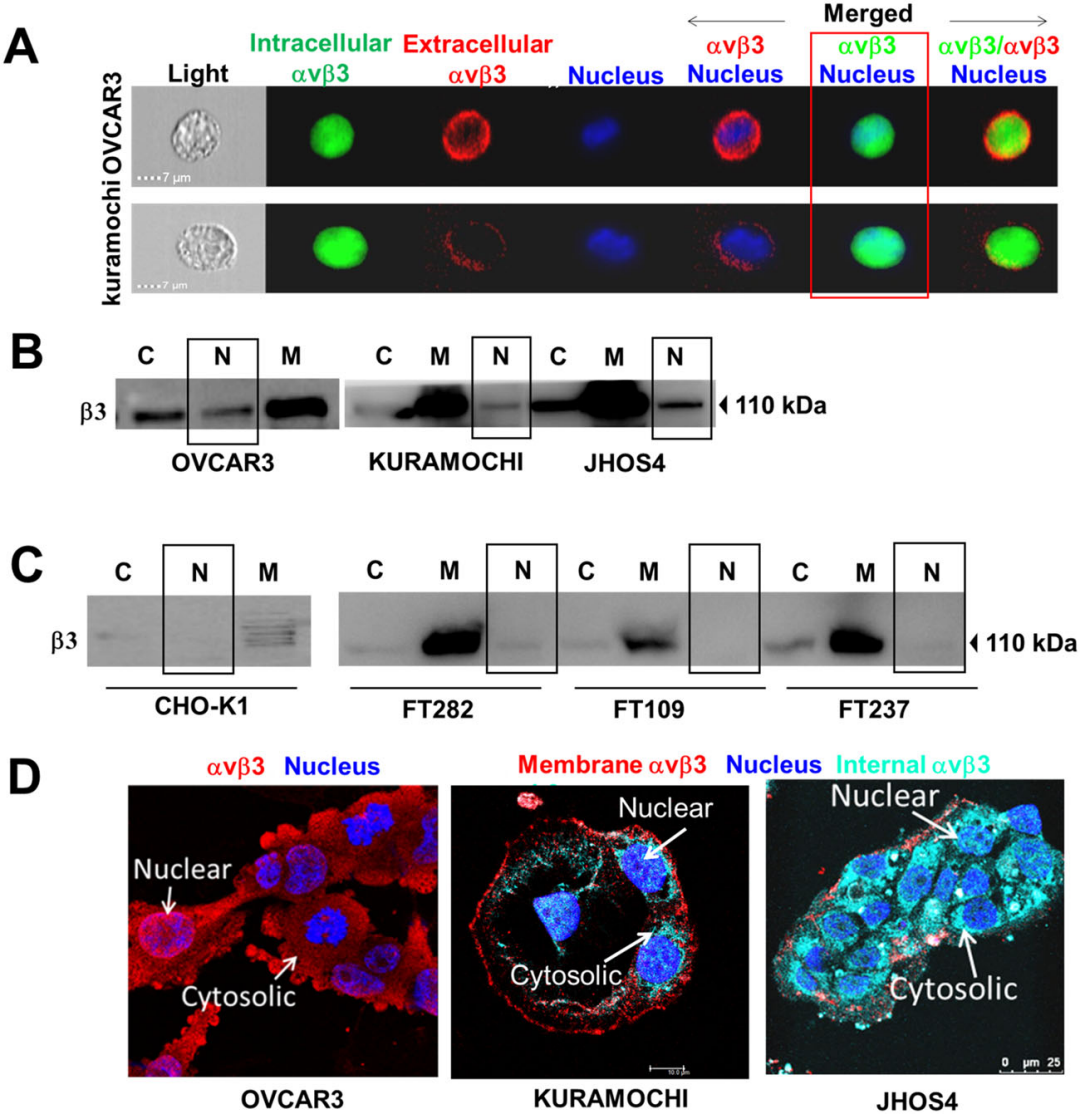
Nuclear αvβ3 in HGSOC cells. Extracellular and intracellular integrins were analyzed by **a** ImageStream of HGSOC cells (1 × 10^5^) stained for membrane (red, PE-labeled) and intracellular αvβ3 (green, NL493-labeld). The nucleus was stained blue with Hoechst. 20× magnification images of separate and merged channels are shown. **b**, **c** Cellular protein fractions from 10 × 10^6^ HGSOC cells, normal ovaries (CHO-K1) and fallopian tube cell lines (FT282, FT109, and FT237) were extracted by Fraction PREP^TM^ Cell Fractionation kit. Proteins were separated using 10% polyacrylamide gels, and analyzed by WB with antibodies against β3. A representative blot of at least three independent repeats is shown. C cytosolic fraction, M membrane fraction, N nuclear fraction. Loading controls and fraction purity are presented in Supplementary Fig. 1B–D. **d** Confocal microscopy in OVCAR3 (active-αvβ3 in red, NL557), KURAMOCHI and JHOS4 (extracellular integrin in red, NL557; intracellular integrin in turquoise, BP680). In all images, a single 0.5 μm optical section is presented using LeicaSP5 confocal microscopy, with a single cut images using 63× objective. Cells (1 × 10^5^) were seeded in glass bottom 6-well plates. The nucleus was stained blue with Hoechst. Negative isotype IgG staining was validated (Supplementary Fig. 1F).

To study the integrin localization we used an antibody that recognizes the αvβ3 integrin dimeric form (clone LM609) and double stained the cells for membrane integrin in red and intracellular integrin in green. The cells were visualized using the ImageStream flow cytometer, an advanced technology acquiring both integrated fluorescence signals and high quality fluorescence images, combined with multiparametric cell analyses. This allowed us to identify atypical αvβ3 nuclear localization in OVCAR3, KURAMOCHI, and JHOS4 (Fig. 1a), HGSOC cell lines which highly resemble the genetic profile of the human disease^12^. Moreover, a similarity score, which measures the overlap between the pixel intensities of paired fluorescent channels in a single image, was calculated by regression analysis using the Pearson’s correlation coefficient. A similarity score above a cutoff >1.5 (Supplementary Fig. 1A) confirmed positive correlation between the green (intracellular αvβ3) and the blue (cell nucleus) channels in OVCAR3 and KURAMOCHI cells (1.95 ± 0.37 and 1.95 ± 0.34, respectively) and no similarities between the remaining channels. Further verification was provided by WB analysis of cytosolic, membrane, and nuclear proteins. Anti-β3 monomer antibody was used as a proxy for the integrin dimer^13^, as in nonplatelet cells it complexes only with the αv monomer^7^. Comparable with the ImageStream results, β3 integrin was identified, besides the membrane and cytosol, also within the nucleus of HGSOC cells (Fig. 1b). Notably, the nuclear integrin was negligible (Fig. 1c) in normal Chinese hamster ovary cells (CHO-K1), as well as in immortalized human fallopian tube cells (FT282, FT109, and FT237), considered the cell-of-origin of HGSOC^2^. Fraction purity was confirmed for all models (Supplementary Fig. 1B–D) and normalized band quantifications in Supplementary Fig. 1E. Confocal microscopy images of a single 0.5 μm optical section provided the final confirmation for a nuclear αvβ3 in a subpopulation of OVCAR3 cells (Fig. 1d, left panel) using a functionally active αvβ3 epitope antibody^14^. Notably, no nuclear integrin was observed in OVCAR3 cells during mitosis, as was shown before for another integrin member which resides underneath or close to the nucleus during cell division^15^. Similar nuclear localization was observed in KURAMOCHI and JHOS4 using an antibody for the integrin dimeric form (Fig. 1d, middle and right panels, respectively). Negative controls are depicted in Supplementary Fig. 1F. The staining pattern suggests integrin interchromatin clustering, probably in nuclear speckles.

**Fig. 2:**
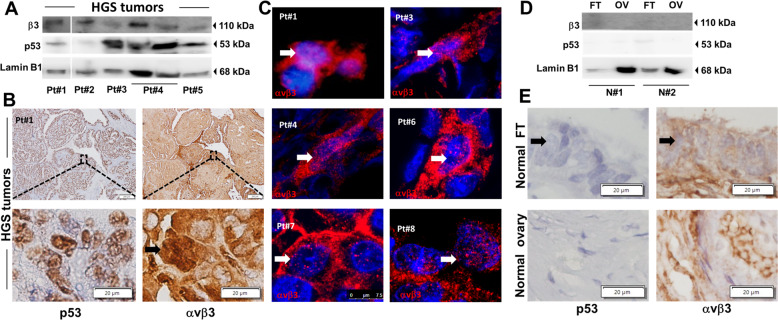
Nuclear αvβ3 localization in HGSOC tissues from patients. **a** Nuclear protein fractions from HGSOC tissues were extracted using Fraction PREP^TM^ Cell Fractionation kit, separated using 10% polyacrylamide gels, and analyzed by WB with antibodies against the β3 integrin monomer. p53 antibody was used to verify tumor region and Lamin B1 antibody to confirm nuclear fraction. Tumor region for Pt#2 (p53 deleted) was confirmed using PAX8 antibody (data not shown). Representative blot of at least three independent repeats is shown. Skipping lanes are clearly marked by a white line. **b** IHC staining of p53 (left panel) and αvβ3 dimer (right panel) in a representative FPPE HGSOC tumor tissue from patient #1. IHC images were obtained by a microscope equipped with a camera using Cell^A Olympus software imaging, 4× objective and 200 µm scale bars for upper panels and 40× objective with 20 µm scale bars for lower panels. Black arrow indicates cells with nuclear αvβ3 (**c**) αvβ3 IF staining in FPPE HGSOC human tumor tissues from six patients (patient #1, #3–4, and #6–8). IF images for patient #1 were generated using IF microscopy with ×40 objective, while the remaining patients were imaged using LeicaSP5 confocal microscopy. Single 0.5 μm optical sections are presented using 63× objective. Hoechst was used to detect the nucleus. White arrows indicate nuclear αvβ3. **d** Nuclear protein fractions from normal fallopian tubes and ovaries were extracted as detailed above and analyzed by WB with β3 antibodies. Lamin B1 antibody was used as a nuclear marker and lack of p53 staining confirms normal tissues. FT fallopian tube, OV ovary. Representative blot of at least three independent repeats is shown. **e** IHC staining for p53 (left panel) and αvβ3 (right panel) in normal fallopian tube and ovary tissues from a healthy control (N#1). 40× magnification. Scale bars: 20 µm. Black arrows indicate serous of the FT.

Next, the existence of nuclear αvβ3 integrin was explored in formalin-fixed paraffin-embedded (FFPE) tumors from nine HGSOC patients (Pt#1–9) and ovarian and FT tissues from eight normal controls (N#1–8). All samples underwent analysis for p53, pax8, or p16^16^, to confirm tumor/normal regions (Supplementary Fig. 2A, B, respectively). Fresh tissues were further collected from five HGSOC patients (Pt#1–5) and two normal controls (N#1–2) and nuclear proteins were extracted. WB analyses confirmed the presence of variable levels of nuclear αvβ3 in HGSOC tumors (Fig. 2a). Fraction purity is presented in Supplementary Fig. 2C and band quantifications in Supplementary Fig. 2D. A parallel immunohistochemistry (IHC) analyses, using an integrin dimer antibody, supported this observation. A representative IHC image displays characteristic nuclear p53 positive tumor cells (Fig. 2b, left panel) with scattered nuclear αvβ3 staining (Fig. 2b, right panel). Additional patients are displayed in Supplementary Fig. 2E. For six tumors IF confocal microscopy images (Fig. 2c), demonstrating nuclear integrin in a subset of tumor cells. Notably, tumor-free (p53 negative) FT serous epithelia from HGSOC patient #1 displayed insignificant integrin staining (Supplementary Fig. 2F). Similarly, lack of nuclear integrin was confirmed in nuclear extracts of normal FTs and ovarian tissues by WB (Fig. 2d) with background membranal αvβ3 staining in the FT serous as well as the ovaries (Fig. 2e). The remaining normal FT samples and ovaries are presented in Supplementary Fig. 2G, H, respectively. Collectively, these results laid the basis that, besides the well-recognized membrane localization, there is a pool of nuclear αvβ3 in human HGSOC cells and tissues.

### αvβ3 traffics to the nucleus in normal integrin-transfected cells

**Fig. 3:**
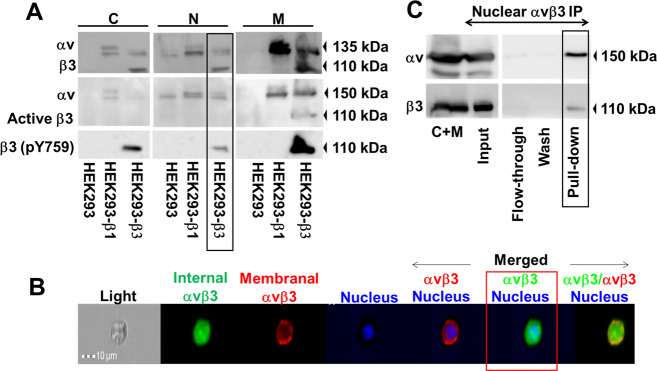
Nuclear αvβ3 localization in integrin transfected HEK293 cells. **a** WB analysis of αv/β3 monomers, ligand occupied β3 and phosphorylated β3 in native and integrin transfected HEK293 cells. Fraction loading and purity is depicted in Supplementary Fig. 3B–D. **b** Extracellular and intracellular integrin was analyzed in αvβ3-HEK293 cells by ImageStream. The cells (1 × 10^5^) were stained for membrane (red, PE-labeled) and intracellular αvβ3 (green, NL493-labeled). 20× magnification images of separate and merged channels are shown. **c** IP of nuclear αvβ3-HEK293 cells (5 × 10^6^) using anti αvβ3-antibody. Immunoblot with β3 or αv monomer antibodies is shown. Negative controls are depicted in Supplementary Fig. 3G–H. c cytosolic fraction, M membrane fraction, N nuclear fraction. Experiments are representative of two independent repeats.

We were next interested to explore nuclear αvβ3 in normal cells forced to express this receptor. HEK293 cells that display endogenous αv but not β3 (αvβ3 negative), were stably transfected with β3 expression plasmid (HEK293β3, αvβ3 positive) or β1 expression plasmid (HEK293β1, αvβ1 positive/αvβ3 negative). Supplementary Fig. 3A depicts αvβ3 membrane level in these cells. Next, equal amounts of cells were collected (Supplementary Fig. 3B) and cytosolic, nuclear, and membrane proteins were extracted. The nuclear fraction was devoid of GRP78, an ER protein extracted with the membrane proteins, excluding cross contamination between fractions (Supplementary Fig. 3C). Additional verification for fraction purity is provided in Supplementary Fig. 3D. WB analysis, using specific antibodies recognizing αv and β3 monomers, confirmed nuclear integrin expression only in the β3-transfected cells (Fig. 3a). Bands quantifications are depicted in Supplementary Fig. 3E. Using two antibodies that recognize active αvβ3 epitopes, CRC54 clone^14^ or the pY759 phosphorylated form^17^, established that the nuclear receptor in the transfected cell model is phosphorylated and functionally active. ImageStream technology further confirmed the existence of nuclear integrin in HEK293β3 (Fig. 3b), with a high similarity score between the green (intracellular αvβ3) and the blue (cell nucleus) channels (1.95 ± 0.3), and no similarities between the remaining channels (Supplementary Fig. 3F). Lastly, IP with an anti αvβ3 antibody that recognizes the integrin dimer (clone LM609) established the presence of both integrin monomers in the nuclear fraction (Fig. 3c). IgG isotype (Supplementary Fig. 3G) and nontransfected cells (Supplementary Fig. 3H) served as negative controls.

### Enrichment of nuclear αvβ3 integrin promotes HGSOC cell proliferation

**Fig. 4:**
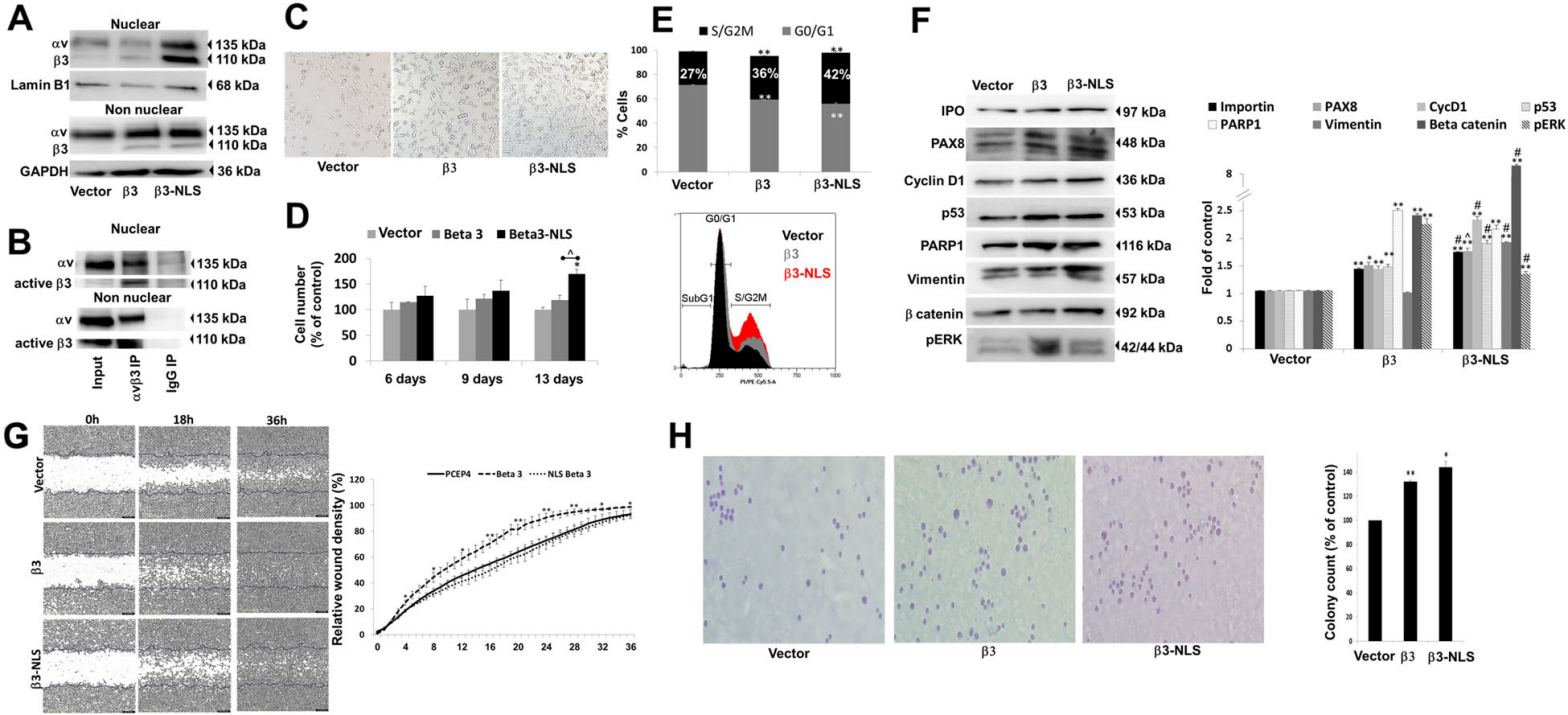
Ectopic expression of nuclear αvβ3 integrin enhances cancer cell proliferation and oncogenic signaling pathways. OVCAR3 HGSOC cells (0.5 × 10^6^/6 well plates) were transfected with an empty vector (PCEP4 plasmid), β3 expression plasmid (β3-PCEP4) or NLS modified β3 vector (β3-NLS-PCEP4) and underwent analysis of **a** Nuclear and nonnuclear protein expression of αv and β3 monomers by WB. Fraction purity is depicted in Supplementary Fig. 4A. **b** Representative immunoblots from OVCAR3 cells transfected with β3-NLS vector following IP from nuclear and non-nuclear fractions using anti-αvβ3 dimer or IgG control antibodies. Protein input and pull down fractions are depicted for αv integrin and active β3 epitopes in the nuclear and non-nuclear fractions. **c** The cells were seeded in triplicates (5000 cells/96 well plates) for 3 days and cell density was imaged (Olympus, model IX71, 4× objective lens) using Cell^A (version 3.1) Olympus software. **d** Cell counts and **e** cell cycle using MACSQuant, Miltenyi Biotec flow cytometer. Percentage of triplicate cells in the S/G2M phase is shown. In the lower panel, a representative cell cycle histogram. **f** Oncogenic signaling pathways in nuclear extracts separated in parallel in three identical WB. Lamin B1 loading control for pERK is shown in Supplementary Fig. 4D, for vimentin, PAX8, and IPO in Supplementary Fig. 4E, for β catenin in Supplementary Fig. 4F and for Cyclin D1, p53, and PARP1 in Supplementary Fig. 4G. Experiments are representative of at least three independent repeats. Quantification of band intensity of the corresponding WB is presented on the right panel as ratio of target protein, normalized to Lamin B1. Densitometry is expressed as percentage compared with the vector-transfected group (considered as 100%). Values are means ± STE. **g** Analysis of wound healing closure for up to 36 h in the various transfected cells (4.5 × 10^4^/96 well plates) using the IncuCyte ZOOM™ real time live cell imaging system. Graph of the relative wound density over time in the various cells is shown on the right panel. **h** Representative images of PCEP4, β3, and NLS-β3 transfected OVCAR3 cells analyzed by soft agar colony formation assay. After staining with 0.05% crystal violet, images were captured (Olympus, model IX71, 20× objective).The graph (right panel) depicts relative colony number (means ± STE). All experiments were repeated at least twice in triplicates. *p < 0.05; **p < 0.005 for comparing the level in the β3 and β3-NLS cells relative to that of vector transfected cells. ^*p* < 0.05; ^#^*p* < 0.005 for comparing the level in the β3-NLS cells relative to that of the β3 transfected cells.

We next explored the functional role of the nuclear αvβ3 in HGSOC. The possibility of mutating putative NLS motifs in the αv and β3 monomers was excluded due to weak prediction scores, identified using bioinformatics tools (data not shown). Loss-of-function approach was also disqualified, as it would have significantly altered the membrane integrin expression and the cell phenotype. In addition, the relatively low amount of αvβ3 in the nucleus makes it challenging to study the actual nuclear function and distinguish it from that of the membrane. To overcome these limitations, we set to ectopically enrich αvβ3 amount inside the nucleus. For that, we modified a β3 expression plasmid (PCEP4 backbone) with NLS motif at the C terminal end of the mRNA sequence (β3-NLS-PCEP4). OVCAR3 cells, chosen as the proof of concept HGSOC model, were transfected with PCEP4, β3-PCEP4, or β3-NLS-PCEP4 vectors and stable lines were generated, following antibiotics selection. Enhanced nuclear integrin expression was confirmed in the OVCAR3−β3-NLS cells (Fig. 4a, upper panel), compared to cells transfected with native β3 or empty plasmids, while membrane integrin expressions were comparable between the transfected cells (Fig. 4a, lower panel). Fraction purity is depicted in Supplementary Fig. 4A. Moreover, by performing IP, the NLS-modified β3 vector was shown to associate with the native αv monomer and to present an active β3 integrin conformation in both membrane and nuclear fractions in the transfected cells (Fig. 4b). In contrast, actin and talin, two cytoskeletal proteins which may physically connect between the membrane integrin and the nuclear lamina, were not bound to the αvβ3 in the nuclear fractions of OVCAR3-β3-NLS cells and HEK293b3 transfected cells (Supplementary Fig. 4B). This suggests that the identification of the nuclear integrin may not be attributed to cytosolic chemical annexation. Therefore, this system is a valid platform for deciphering the potential contribution of the nuclear αvβ3 in HGSOC.

First, the various transfected cells were equally seeded in triplicates and assessed after 3 days by microscopy. Results showed significantly higher cell density in OVCAR3 cells enriched with the nuclear αvβ3 compared to cells transfected with an empty vector or the native integrin (Fig. 4c). Continuous growth for up to 13 days confirmed this phenotype by flow cytometry analyses of absolute cell counts (Fig. 4d), with a 1.67-fold increase at study end in the NLS-integrin transfected cells compared to empty and β3 vectors (*p* < 0.005). A similar trend in the percentage of cells undergoing DNA synthesis and mitosis (S/G2M phase) was demonstrated by cell cycle analysis in the β3-NLS transfected cells (Fig. 4e). A representative cell cycle histogram is shown in the lower panel. Next, nuclear proteins were extracted from the various transfected cells. Fraction loadings are presented in Supplementary Fig. 4C–F. Representative Western blots of an array of signaling pathways are shown in Fig. 4f and quantifications is shown on the right panel. Relative to empty-vector transfected cells, an increase in importin beta (IPO), involved in cargo delivery into the nucleus, was shown in β3-transfected cells (1.3-fold, *p* < 0.005) and more so in β3-NLS cells (1.66-fold, *p* < 0.005). Similarly, higher level of PAX8 and cyclin D1, both involved in cell proliferation, were shown in β3 overexpressing cells (1.4-fold and 1.37-fold, respectively). This effect was significantly more pronounced in the β3-NLS cells with a 1.67-fold increase for PAX8 and a 2.2-fold increase for cyclin D1 (*p* < 0.005). Moreover, the level of OVCAR3 gain-of-function p53 mutant (R248Q), was induced in the β3-transfected cells by 1.4-fold and more potently in the β3-NLS transfected cells (1.8-fold, *p* < 0.005). Nuclear vimentin and β catenin, both associated with epithelial to mesenchymal transition (EMT), were also significantly induced in the β3-NLS cells (1.8-fold and 8.3-fold, respectively, *p* < 0.005), compared to cells transfected with the β3 vector (0.96-fold and 2.3-fold respectively). In contrast, the β3-tranfected cells displayed greater induction in PARP1 (2.4-fold) and phosphorylated ERK (pERK, 2.2-fold), compared to the β3-NLS cells (2-fold and 1.2-fold, respectively, *p* < 0.005), suggesting that this signaling is primarily regulated via the membrane integrin. The contribution of the nuclear integrin to ovarian cancer cell migration was next assessed by wound-healing assay (Fig. 4g). The relative wound density is depicted on the right panel. While β3 accelerated wound healing closure, the NLS-β3 cells displayed rate comparable with that of the control cells, suggesting, besides dominance of the membrane integrin, that the nuclear integrin may inhibit cell migration. Nonetheless, soft agar colony formation (Fig. 4h) remained intact in cells expressing the NLS-modified integrin, signifying that nuclear enrichment did not hinder the tumorigenic potential of the malignant cells.

### The nuclear αvβ3 complexes with central cancer-relevant proteins

Finally, we aimed to elucidate the biological function of the nuclear integrin through identification of potential interacting proteins. To that end, we performed αvβ3 Co-IP for nuclear extracts of cells overexpressing the integrin (HEK293β3) and the three HGSOC cell lines (OVCAR3, JHOS4, and KURAMOCHI). The integrin dimer was pulled-down using αvβ3 antibody (clone LM609) by magnetic beads. Co-IP performed without the primary antibody (beads only) or in integrin negative cells (HEK293), served as negative controls and was used to quantify the assay background. Nuclear proteins were analyzed by LC-MS/MS and considered nuclear integrin-associated (unique) when having greater than 4-fold binding from Co-IP with beads and complete absence in integrin negative cells.

**Fig. 5:**
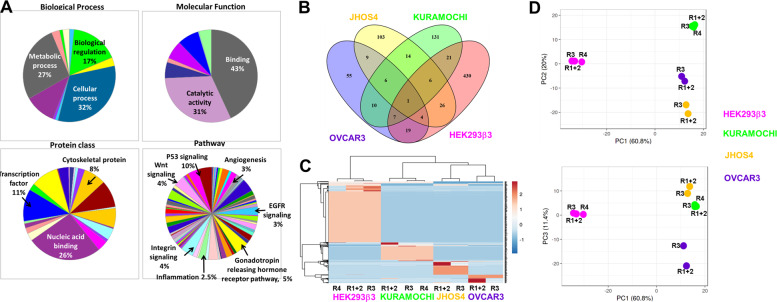
Proteomics analysis of the co-IP results in integrin-transfected HEK293 cells and HGSOC cell lines. **a** Pie charts results using PANTHER tool analyzing biological process, molecular function, protein class and pathways related to the identified proteins. Percentage of the main subcategories is depicted. **b** Shared/unique proteins (absolute number) between HEK293β3, pink; OVCAR3, purple; JHOS4, yellow and KURAMOCHI, green using the Venny diagram tool. **c** Unsupervised hierarchical clustering, using the ClustVis web tool, of the integrin-bound proteins, delineating the four major cell line groups (same color codes as in **b**). Rows are centered. Unit variance scaling is applied to rows. Both rows and columns are clustered using correlation distance and average linkage. **d** Principal component analysis (PCA) of the four cell lines. Unit variance scaling is applied to rows, SVD with imputations were used to calculate principle components. On the upper panel, *X* and *Y* axis show principle component 1 and 2 that explain 60.8% and 20% of the total variance, respectively. On the lower panel, component 1 and 3 that explain 60.8% and 11.4% of the total variance, respectively.

Proteomics analysis identified a total of 843 αvβ3-associated proteins, of which approximately 514 in HEK293β3 cells, 111 in OVCAR3, 169 in JHOS4 and 196 in KURAMOCHI (Supplementary Table 1). Notably, the majority of proteins displayed complete lack of assay background, providing high level of confidence in results validity. Interaction network between the proteins in the various cells (Supplementary Fig. 5), generated using the STRING tool^18^, confirmed protein–protein interaction (PPI) enrichment *p*-values of <1.0e−16 for HEK293β3 and JHOS4, <5.3e−14 for OVCAR3, and <3.3e−10 for KURAMOCHI. This indicates that the protein network has significantly more interactions among themselves and is biologically connected as a group, than what would be expected for a random set of proteins of similar size drawn from the genome. Using the PANTHER tool^19^ main subcategories of the nuclear αvβ3-bound proteins are presented in pie charts (Fig. 5a). Biological processes mainly involve metabolic, cellular and biological regulation (27%, 32%, and 17%, respectively), molecular function is largely via protein binding (43%) and catalytic activity (31%), while protein class is commonly of nucleic acid binders (26%) and transcription factors (11%). Multiple signaling pathways were associated with the nuclear integrin, including p53 (10%) and Wnt (4%). Using Venny diagram tool^20^, we assessed the shared versus unique proteins in the different cells (Fig. 5b). While HEK293β3 displayed only 16% similarity with the HGSOC protein panel, in the HGSOC cells between 33–50% of the proteins were shared with at least one other cell model. Statistical analyses of the proteomics data indicated significant difference between the three HGSOC cells (*p* < 0.001), and more profoundly between these cells and HEK293β3 (*p* < 2.7 × 10^–22^). For visualizing clustering of multivariate data, the ClustVis web tool was used^21^. Heatmap results of unsupervised hierarchical clustering on the basis of the relative expression of the integrin-bound proteins in the four cell groups, clearly delineated that the HGSOC cells cluster closer compared to the integrin transfected normal cells (Fig. 5c). Principal component analysis (PCA) further confirmed the segregation of the HGSOC clusters from that of the HEK293β3 cells (Fig. 5d). This corresponds with the mutual disease origin shared by the HGSOC lines. Notably, in agreement with previous published works^12^, both analyses suggested a closer similarity between JHOS4 and KURAMOCHI compared to OVCAR3.

Lastly, we focused on 57 integrin-bound proteins that were shared between the various HGSOC cells (Table 1). Seventy-seven percent of these proteins were present in KURAMOCHI and 67% in JHOS4 and OVCAR3. In contrast, only 30% of these proteins were eluted with the nuclear integrin in HEK293β3, although these cells express significantly higher levels of αvβ3 and display superior number of integrin-bound proteins. This further accentuates the distinction observed between HEK293β3 and the HGSOC cells panel using cluster analysis methods. According to the gene ontology (GO), the shared proteins belong to ten categories of biological processes. These include eight proteins involved in cell cycle and mitosis, among which Cullin-5 (CUL5) was the only protein that was commonly eluted in both the transfected cells and the entire HGSOC panel. We also identified proteins associated with apoptosis, such as CCAR1 and RMDN3, only in the HGSOC cell models. Notably, the nuclear integrin was bound to proteins known to be complexed with the membrane integrin^22^, including the cytoskeletal proteins Filamins (FLNA and FLNC), palladin (PALLD), and RAS-GTPase-activating-like protein (IQGAP1). Similarly, integrin linked kinase (ILK) and Talin 1 (TLN1) were identified, although only in specific cell models. Collectively, this indicates that at least some of these canonical proteins also interact with αvβ3 within the nuclear compartment. In addition, a large group of proteins regulating both transcription and translation were associated with the nuclear αvβ3, including the integrator complex subunit 2 (INTS2) and the eukaryotic translation initiation factor 5B (EIF5B). Lastly, several proteins involved in RNA, vesicles and protein transport, were identified, for example the translocation protein SEC62. Additional proteins facilitating in-and-out nuclear trafficking, including exportin, importins, clathrins, and nexins were also integrin bound, although unique subunits were identified in the various cell models. This, combined with the observed importin induction in the NLS-modified integrin cells, proposes a trafficking mechanism for the nuclear integrin. Collectively, the nuclear αvβ3 interactome suggests potentially novel moonlighting activities for this receptor.

**Table 1:**
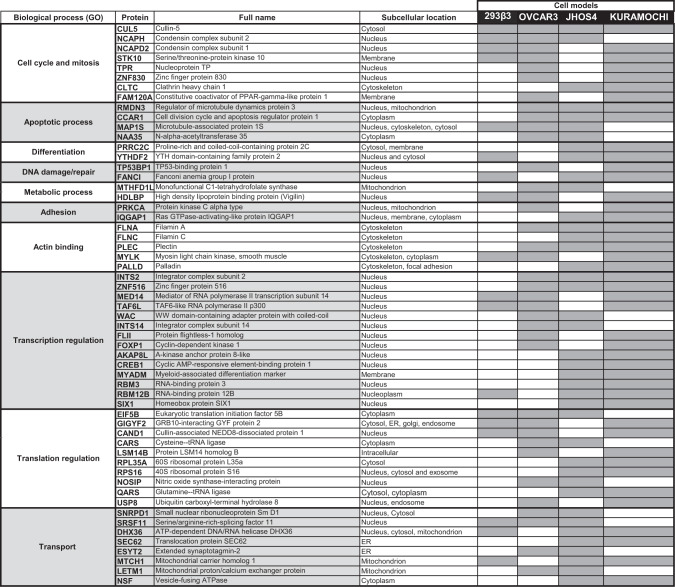
Shared nuclear αvβ3-integrin bound proteins from in the various cell models.

The table depicts different categories of biological processes according to Gene Ontology (GO), the protein short and full names, subcellular location and absence (white color) or presence (gray color) in the various cells.

## Discussion

The presence of cell surface receptors in the nucleus was recognized decades ago, however, this research field is still relatively neglected in cancer in general and ovarian cancer in particular. Although integrins are known to recycle to and from the plasma membrane^23^, work on nuclear integrin trafficking is scarce. Two reports suggested nuclear trafficking of the αv or β4 integrin monomers in cancer cells^24,25^. This trafficking, however, did not involve the full receptor form and was evident only following specific stimuli. In this work, we identified atypical nuclear localization of the full αvβ3 integrin receptor in HGSOC cells, but not normal FT cells and tissues. Since mutated FT epithelium is recognized as the source of HGS carcinoma^2^, we postulate that αvβ3 trafficking into the nucleus may take part in this transformation. The nuclear αvβ3 was phosphorylated in cells ectopically expressing the integrin, suggestive of an active receptor conformation^17^. Identification of a nuclear αvβ3 reservoir challenges the prevalent conception that the setting in which this receptor exerts its pleiotropic actions is exclusively at the cell membrane and proposes protein moonlighting functions.

To lay a clear foundation for the functional role of the nuclear αvβ3, we generated, using NLS-modified β3 integrin vector, HGSOC cells with stably enhanced nuclear integrin expression. This cell platform established, by multiple complementary methods, that the nuclear αvβ3 increased cancer cell proliferation and activation of a host of oncogenic proteins. Among which, elevation in the gain-of-function R248Q mutant p53^26^, cyclin D1 and β catenin, known to transcriptionally regulate cyclin D1^27^, as well as vimentin which like β catenin is involved in EMT and ovarian cancer progression^28^. PAX8, a lineage marker of the fallopian tube, shown to be associated with enhanced ovarian cancer proliferation^29^, was also induced. In addition, NLS-modified integrin cells exhibited induction in importin beta, a nuclear import protein^30^, signifying a mode of nuclear delivery. Our results are in agreement with reports on oncogenic pathway activation by retrograde nuclear trafficking of other transmembrane proteins^4^. In contrast, activation of ERK and cell migration were observed only in cells expressing the native αvβ3, suggesting regulation primarily via the membrane integrin, roles that are well-established^31,32^. Collectively, although membrane integrins are known to govern signal transduction pathways^22^ and nuclear alterations^33^, our current work accentuates the critical novel role these receptors may play directly from within the nuclear compartment.

Using proteomics analysis, a network of αvβ3-interacting proteins was revealed in the various cell models. Notably, a significantly higher number of proteins were identified in the integrin-transfected cells, suggesting that this platform facilitates identification. HGSOC cells and αvβ3-transfected normal cells distinctly cluster, proposing that the protein complexes are cell type and/or disease-related. Among the nuclear-integrin-complexed proteins, we identified a group involved in cell cycle and mitosis. These include CUL5, a G2/M blockade protein that is downregulated in many tumor types, including ovarian cancer^34^, resulting in substantial proliferation. Interestingly, this protein was shown to target β1, another integrin family member, for degradation^35^ and to regulate focal adhesion dynamics in epithelial cells^36^. It remains to be studied whether complexing of CUL5 with the nuclear αvβ3 participates in its turnover and tumor-promoting actions. Several proteins involved in apoptosis were also nuclear integrin-bound, including CCAR1, a key regulator of chemotherapy-induced apoptosis^37^. CCAR1 correlates with ovarian cancer progression-free-survival^38^ and interacts with the nuclear estrogen receptor α, an ovarian cancer relevant growth hormone, as well as with SRC^39^, which canonically complexes with the membrane αvβ3 integrin. The second apoptotic-related protein, RMDN3, also known as PTPIP51, is highly expressed in several cancers and regulates and interconnects with central oncogenic signaling pathways^40^. Our work is the first to implicate a role for both proteins in complexing with the nuclear integrin in HGSOC.

Furthermore, we have unexpectedly identified proteins that commonly complex with the membrane αvβ3^22^, such as Filamins, Palladin, and Plectin, to be physically bound with this receptor inside the nucleus. Similarly, ILK, known to take part in focal adhesions^41^ was observed, although only in the integrin transfected cells. Nuclear trafficking of these proteins was previously reported in several malignancies^42,43^, providing support for our findings. Collectively, this suggests that nuclear translocation of integrin adherent structures may mediate their intranuclear effects, either through classical functions or via new mechanisms. We also identified key proteins involved in transcription and translation regulation bound with the nuclear integrin. These include INTS2, which is highly mutated in HGSOC^44^ and EIF5B, which is a critical regulatory pro survival node in several aggressive cancers^45^. Another central regulator of transcription, the Histone acetyltransferase p300 protein (EP300), was bound to the nuclear αvβ3 in the integrin transfected cells and one of the HGSOC cell models. This specific protein was reported before in OVCAR3 cells to complex, following thyroid hormone stimulation, with the nuclear αv monomeric form, but not with the full receptor as observed by us^24^. Lastly, several proteins involved in RNA, vesicles and protein transport were identified with the nuclear integrin. For example, the translocation protein SEC62, which is located at frequently amplified chromosomal region in numerous human cancers, including ovarian^46^. With regards to transport, several proteins facilitating in-and-out nuclear trafficking, including exportin, importins, clathrins, and nexins^4,30^ were nuclear integrin bound. Specifically, importins and exportins act by binding the cargo protein via nuclear localization signal (NLS) motif^47^ and signify a potential trafficking mechanism for the nuclear integrin.

To conclude, we have identified a nuclear αvβ3 integrin reservoir in ovarian cancer with a proposed role and interactions that may be distinct from its plasma membrane actions. This unique biological mechanism, which has been largely overlooked, adds to the multifaceted activities reported for αvβ3 and may lead to improved understanding of the molecular basis of ovarian cancer with a potential impact on other malignancies.

## Materials/Subjects and methods

### Antibodies

αvβ3 dimer (clone LM-609), αv monomer, Na^+^K^+^ ATPase α-1 (Merck Millipore, Darmstadt, Germany); β3 monomer (Santa Cruz Biotechnology, Dallas, TX, USA), functionally active β3 (clone CRC54); phosphorylated β3 (p-Y759 β3), importin beta, Lamin B1, GRP78 (Abcam, Cambridge, MA, USA); phospho-PI3K p85 (Tyr458)/p55 (Tyr199), β-catenin, vimentin, gapdh, cyclin D1, PARP-1, β Tubulin (Cell Signaling, Leiden, The Netherlands); Horseradish peroxidase (HRP) p53 (R&D systems, Abingdon, UK), PAX8 (Proteintech, Manchester, UK). For IHC and IF of FFPE tissues, αvβ3 integrin dimer (Abbiotec, San Diego, CA, USA), p53 (Novacastra, Wetzlar, Germany), PAX8 (Cell Marque, Rocklin, CA, USA) and p16 (Ventana, Tucson, Arizona, USA). Secondary HRP-IgG, goat antiRabbit IgG, goat antiMouse IgG (Jackson ImmunoResearch Laboratories, West Grove, PA, USA); Mouse IgGκ light chain binding protein-CFL680 (Santa Cruz Biotechnology); DyLight 550 conjugated Donkey antiRabbit antibody (Bethyl Laboratories Inc., Montgomery, TX, USA); IgG- NL493, Donkey antiMouse IgG -NL557 (R&D systems).

### Cell cultures

HGSOC and FT282/FT237/ FT109 cells were a kind gift from Dr. Ruth Perets (Rambam Medical Center, Haifa, Israel) and β3/β1 HEK293 from Prof. Coll (University of Grenoble, France and CHO-K1 from Prof. Philippe Clézardin (University of Lyon, Lyon, France). OVCAR3, KURAMOCHI, Chinese hamster ovaries (CHO-K1) and HEK293 were grown in full RPMI1640, JHOS4, and FT’s in DMEM F-12 and β3/β1 HEK293 in high Glucose DMEM (Biological Industries, Beit Haemek, Israel). STR/mutation profiling was used for authentication and mycoplasma was screened periodically.

### Human tissue samples

Fresh/archived tissues were collected from nine HGSOC patients at diagnosis undergoing primary debulking surgery and eight nononcological patients undergoing laparotomy/ultrasound guided paracentesis at the Obstetrics and Gynecology Department, Meir Medical Center, Israel. Human tissues were collected with informed consent in compliance with the Institutional Review Board approval, in accordance with the Declaration of Helsinki.

### Immunohistochemistry

Four micron sections were cut from FFPE tissue blocks at the Pathology Department, Meir Medical Center, Israel and stained on a Ventana Benchmark XT automatic stainer (Ventana).

### IF confocal microscopy

Cells were seeded for 24 h (1 × 10^5^ cells/24 well glass bottom plates). OVCAR3 cells were fixed (4% PFA), permeabilized (0.2% Triton × 100) and incubated with 10 μg/ml (1ː100) αvβ3 NL557-labled antibody. JHOS4/KURAMOCHI were incubated with NL557-labeled αvβ3 antibody, fixed, permeabilized (0.2% Triton × 100), blocked (1% BSA) and analyzed for intracellular αvβ3 by incubation with BP680-labeled αvβ3 antibody. For IF staining of FFPE tissues, we performed antigen retrieval (Ventana Benchmark XT automatic stainer), manual blocking (3% BSA), incubation overnight with αVβ3 antibody and staining with secondary antibodies. Nucleus was stained with Hoechst (33342, molecular probes, Eugene, OR, USA). Tissues/cells were analyzed by Leica SP5 confocal microscopy (Leica, Mannheim, German), using 20×/40×/63× objectives. In all images, a single 0.5 μm optical *z*-plane section is presented.

### Protein extraction

Protein fractions were extracted using Fraction PREP ^TM^ Cell Fractionation kit (BioVision, Milpitas, CA, USA), Nuclear Extraction Kit (Abcam, Cambridge, MA, USA) or Nuclear Complex Co-IP kit (Active Motif, La Hulpe, Belgium).

### Western blot

Proteins were separated and analyzed as detailed before^48^. Bands were measured by Las3000 imaging system and analyzed by Multigauge v3.0 software (Fujifilm Life Science, Tokyo, Japan).

### Flow cytometry

Absolute cell counts and cell cycle were performed using MACSQuant flow cytometer (Miltenyi Biotec, Bergisch Gladbach, Germany), as detailed before^48,49^.

### ImageStream technology

Amnis ImageStream^®X^ Mark II multispectral imaging flow cytometer (Amnis Corporation, Seattle, WA, USA) was used. For αvβ3 membrane expression, 5 × 10^5^ cells were incubated with 10 μg/ml PE-labeled αvβ3 dimer antibody, fixed (4% PFA), permeabilized (0.2% Triton), washed and analyzed for intracellular αvβ3 by incubation with 10 μg/ml NL493-αvβ3 antibody. IgG was used as negative isotype control.

### Co-IP

Nuclear proteins from HEK293β3 (5 × 10^6^ cells), OVCAR3, JHOS4 and KURAMOCHI (~100 × 10^6^ cells each) were separated and αvβ3 dimer antibody was used for pull-down using mMACS kit and protein G magnetic beads (Miltenyi Biotec). 3–5 independent biological replicates were conducted. Co-IP from nuclear extracts with beads only, in αvβ3 negative HEK293 cells (5 × 10^6^ cells), or with isotype IgG, served as negative controls.

### NLS-β3 vector construction

Nuclear localization signal sequence (NLS, MDPKKKRKGR^50^) was introduced into β3 PCEP4 vector at the c terminal end of the mRNA byadding 5′ (SapI) and 3′ (SbfI) sites and custom cloning. The final construct was sequenced using PCEP4 EBV-R primer and by Sanger sequencing (outsourced at GENEWIZ, South Plainfield, NJ, USA).

### Stable transfections

OVCAR3 cells were seeded (0.5 × 10^6^/6 well plates) and transfected a day later with the various vectors (2.5 μg) using 7.5 μl of TransIT-2× transfection regent (Mirus, Madison, WI, USA). After 48 h, selection antibiotic (Hygromycin B, Merck Millipore) was added at 0.75 mg/ml to initiate stable cell lines.

### Wound healing assay

IncuCyte ZOOM™ real time live cell imaging system (Essen BioScience, MI USA) was used to calculate the relative wound density within the initially-vacant area at each time point.

### Soft agar colony assay

OVCAR3 cells were transfected with PCEP4/β3/β3-NLS vectors, suspended in 0.6% noble agar (A5431, Sigma Aldrich) in growth medium and plated (2.5 × 10^5^/6 well plates) onto layer of 0.9% noble agar in growth medium. Fresh medium was added twice a week for 4 weeks. Plates were stained overnight with 0.05% crystal violet in 10% Neutral Buffered Formalin (316–155, Thermo Fisher Scientific), washed and colonies were counted using OpenCFU software. Experiments were repeated twice.

### Proteomics analysis

Nuclear**-**αvβ3-eluted proteins were trypsin digested and analyzed independently or as a pool of two extractions using LC-MS/MS on Q Exactive™ HF-X Mass Spectrometer (Thermo Fisher Scientific, Bremen, Germany). Proteins were identified by Discoverer software version 1.4 against the Human sequence using the search algorithms Sequest (Thermo Fisher Scientific) and Mascot search engines. Identified peptides were filtered with high confidence, top rank, mass accuracy, and a minimum of two peptides. High confidence peptides have passed the 1% false discovery rate threshold (Estimated false positives in a list of peptides). Semi-quantification was done by calculating the peak area of each peptide, with an average of the three most intense peptides from each protein. Proteins were considered nuclear integrin-associated (designated as unique) by having greater than 4-fold binding from Co-IP with beads only and complete absence in the negative control cells. Keratins, possible contaminants, were filtered out.

### Protein enrichment analysis and classification

Protein classification was performed using http://www.geneontology.org/^51^ powered by Protein Analysis Through Evolutionary Relationships (PANTHER, http://pantherdb.org)^19^. Protein–protein interaction network was generated using STRING v. 11 (https://string-db.org) with default settings (minimum required interaction score: medium confidence 0.4)^18^ and clustered to a specified Markov clustering inflation parameter of 3^52^. Venny diagram tool (http://bioinfogp.cnb.csic.es/tools/venny) was used to compare shared/unique proteins^20^. ClustVis web tool (https://biit.cs.ut.ee/clustvis) was used for multivariate cluster analysis using PCA and heatmap^21^.

### Statistical analysis

Experiments were repeated independently in triplicates and analyzed by two-sided unpaired or paired *t*-test and/or by ANOVA for multiple comparisons. Significance was determined at *p* < 0.05. Results are presented as mean ± STE.

## Supplementary information

Supplementary Table 1

Supplementary Figure 1–5

## Data Availability

Full proteomics datasets are available from the corresponding author on reasonable request.
