# Supplementary material for: The identification of nuclear αvβ3 integrin in ovarian cancer: non-paradigmal localization with cancer promoting actions
Source: Oncogenesis. 2020 Jul 29;9(7):69. doi: 10.1038/s41389-020-00254-2 (PMC7391722; doi:10.1038/s41389-020-00254-2)
Supplement: Supplementary file 2 — Supplementary Figure 1–5 [file 41389_2020_254_MOESM2_ESM.pdf]

The identification of nuclear αvβ3 integrin in ovarian cancer: non-paradigmal localization with cancer promoting actions

Seraya-Bareket et al.

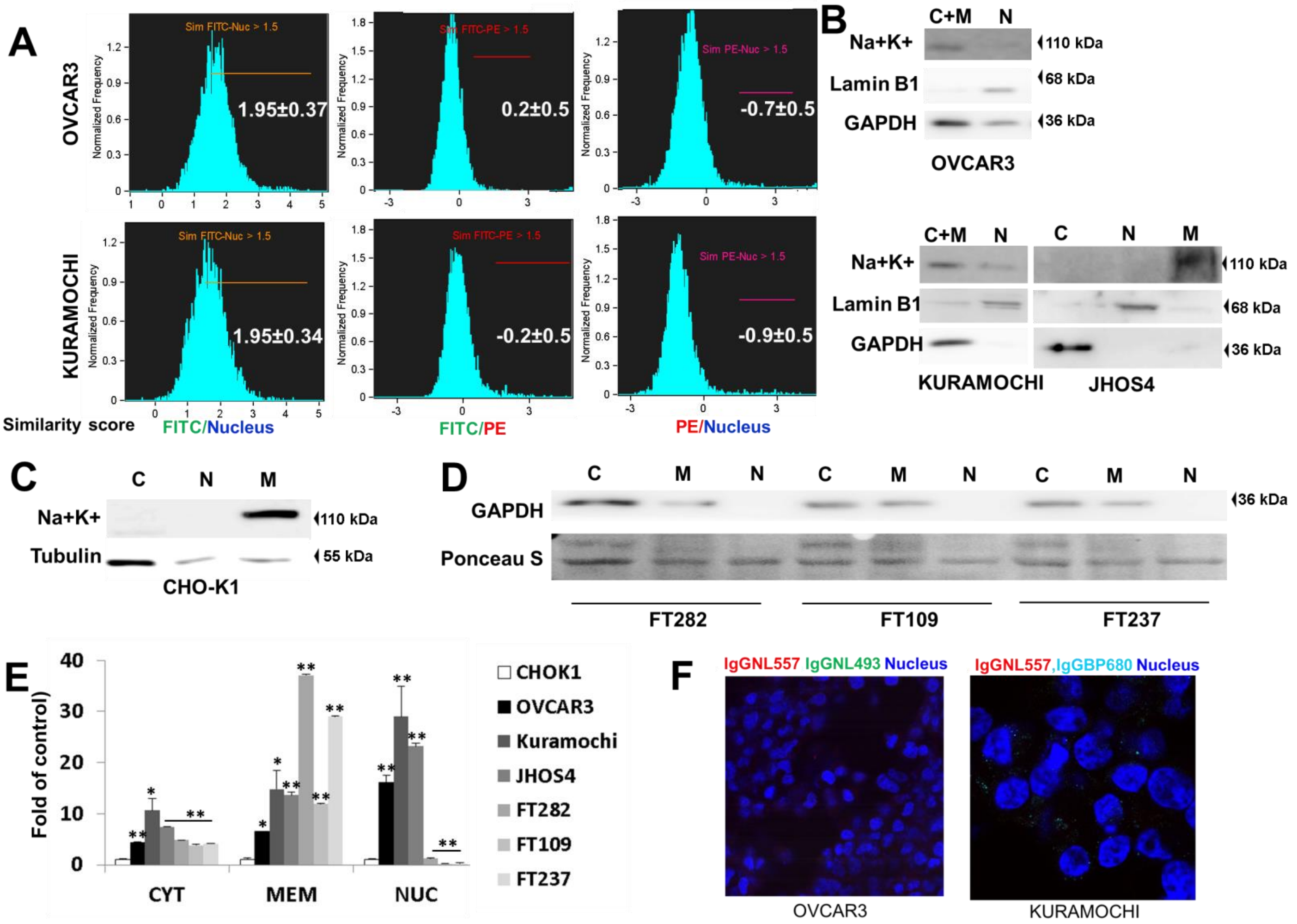

Supplementary Figure 1: (A) ImageStream similarity score histograms for the various channel combinations in OVCAR3 and KURAMOCHI. A high similarity score between the green (extracellular αvβ3) and the blue (cell nucleus) channels is depicted (Average±STDEV) while the other channel combinations present no significant similarity. **(B-D) Controls for Western blots** Western blot analysis of fraction purity and loading controls for the various cell models. Western blots analysis of Na+K+, Lamin B1, GAPDH or tubulin in cytosolic (C), nuclear (N) and membrane (M) protein fractions in **(B)** OVCAR3, KURAMOCHI and JHOS4 HGS cell lines and **(C)** CHO-K1 normal ovary cells **(D)** Loading control (Ponceau S) and fraction purity (GAPDH) in FT282, FT109 and FT237 fallopian tube cell lines. Representative blots are shown. **(E)** Quantification of band intensity of the WB presented in Figure 1 as ratio of target protein, normalized to loading. Densitometry is expressed as percentage compared with CHO-K1 (considered as 100%). Values are means ± STE. **(F) Controls for the confocal microscopy**. IgG isotype controls for the various staining methods in the HGSOC cells. Merged channels are shown.

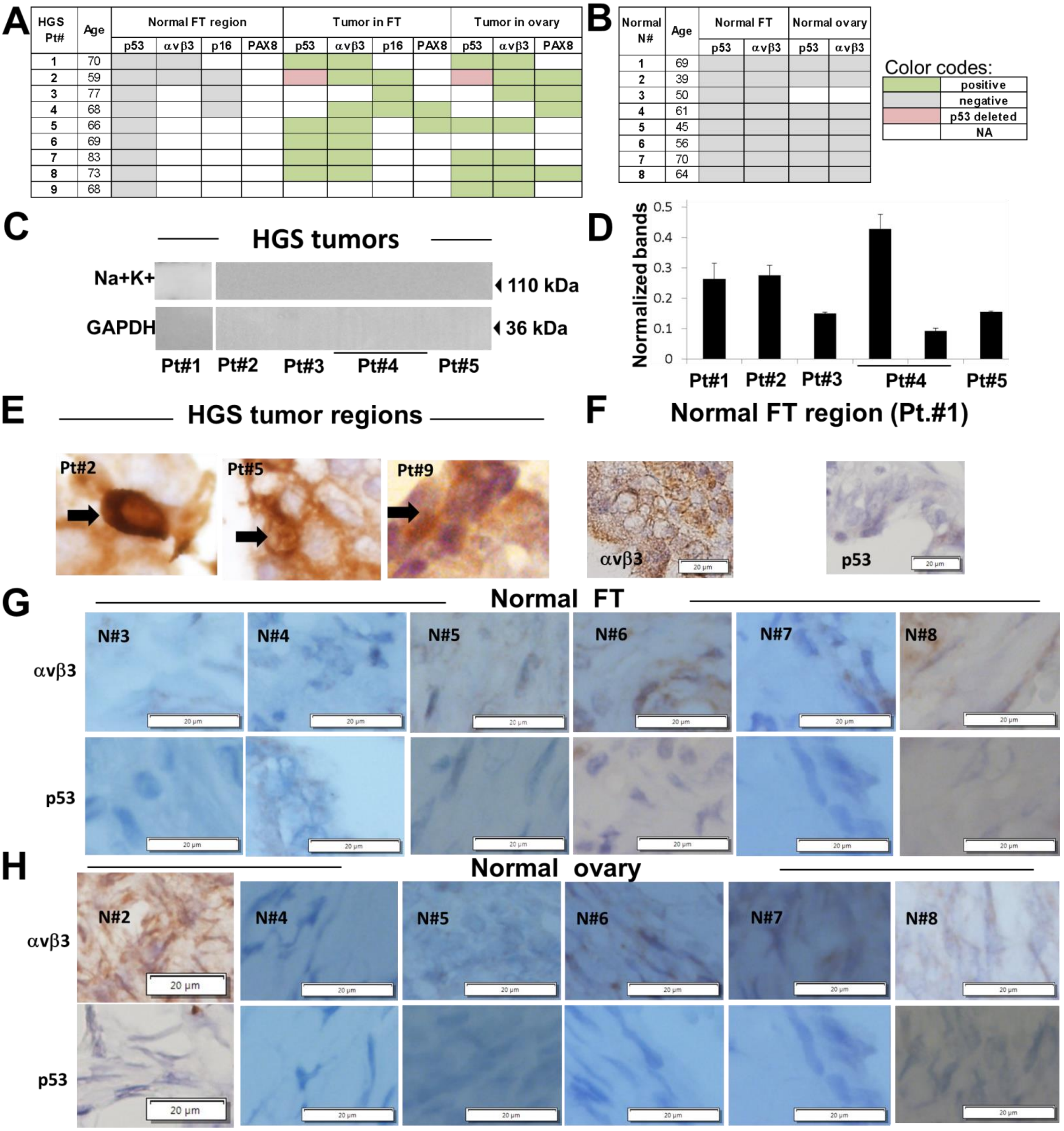

Supplementary Figure 2: Summary of the HGSOC tissue control assessment. (A) IHC analysis for p53, pax8 and p16 in tumor/normal regions in the ovary and FT tissues from HGSOC patients **(B)** FT and ovarian tissues from normal controls assessed for p53. **(C)** Western blots analysis of Na+K+ and GAPDH in nuclear fractions from `patients #1-5. **(D)** Quantification of band intensity of the WB presented in Figure 2A as ratio of target protein normalized to loading. Values are means ± STE. **(E)** FFPE images of HGS ovarian cancer tissues from patients #2, #5 and #9 stained for αvβ3 by IHC. Nuclear integrin is indicated by a black arrow. **(F)** IHC images of tumor free fallopian tube tissue from patient #1 stained negative for p53 and for αvβ3. **(G)** IHC images of normal fallopian tube tissues stained negative for p53 and for αvβ3 **(H)** IHC images of normal ovary tissues stained negative for p53 and for αvβ3. X40 objectives. Scale bars: 20μm.

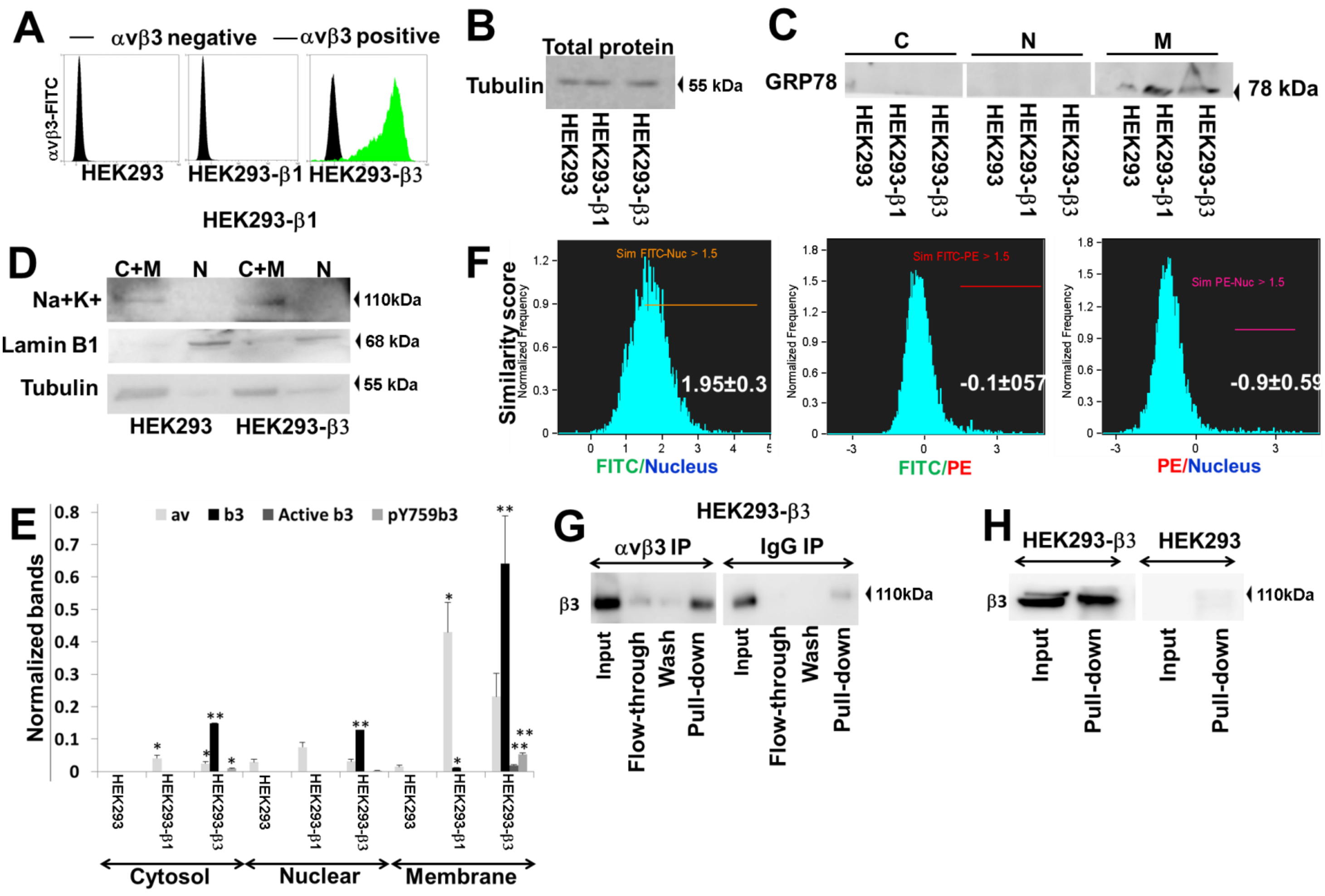

Supplementary Figure 3: Analysis of native and αvβ3 transfected HEK293 cells. (A) Flow cytometry analysis of αvβ3 integrin in non-transfected cells and αvβ1 or αvβ3 transfected cells **(B)** Western blots analysis of tubulin to determine protein extraction from equal amount of cells **(C)** Fraction purity by WB in cytosolic (C), nuclear (N) and membrane (M) protein fractions from native and αvβ3 -transfected HEK293 cells. GRP78, extracted with other membrane proteins, was used an ER marker and tubulin, as a cytosolic marker **(D)** Western blots analysis of Na+K+, Lamin B1 and tubulin in cytosolic and membrane fraction (C+M) and nuclear fraction (N) in HEK293 and HEK293β3 **(E)** Quantification of band intensity of the WB presented in Figure 3A as ratio of target protein, normalized to loading. Values are means ± STE. **(F)** A high similarity score between the green (intracellular αvβ3) and the blue (cell nucleus) channels is depicted (Average±STDEV) while the other channel combinations present no significant similarity in the HEK293β3 cells. **(G)** Representative Western blots from αvβ3 integrin in αvβ3-tranfected HEK293 cells following IP using anti αvβ3 dimer antibody or an isotype control IgG antibody. Protein input, flow-through, wash and pull down fractions are depicted **(H)** Representative Western blots from αvβ3 integrin in αvβ3-tranfected and native HEK293 cells following IP using anti αvβ3 dimer antibody. Protein input and pull down fractions are depicted.

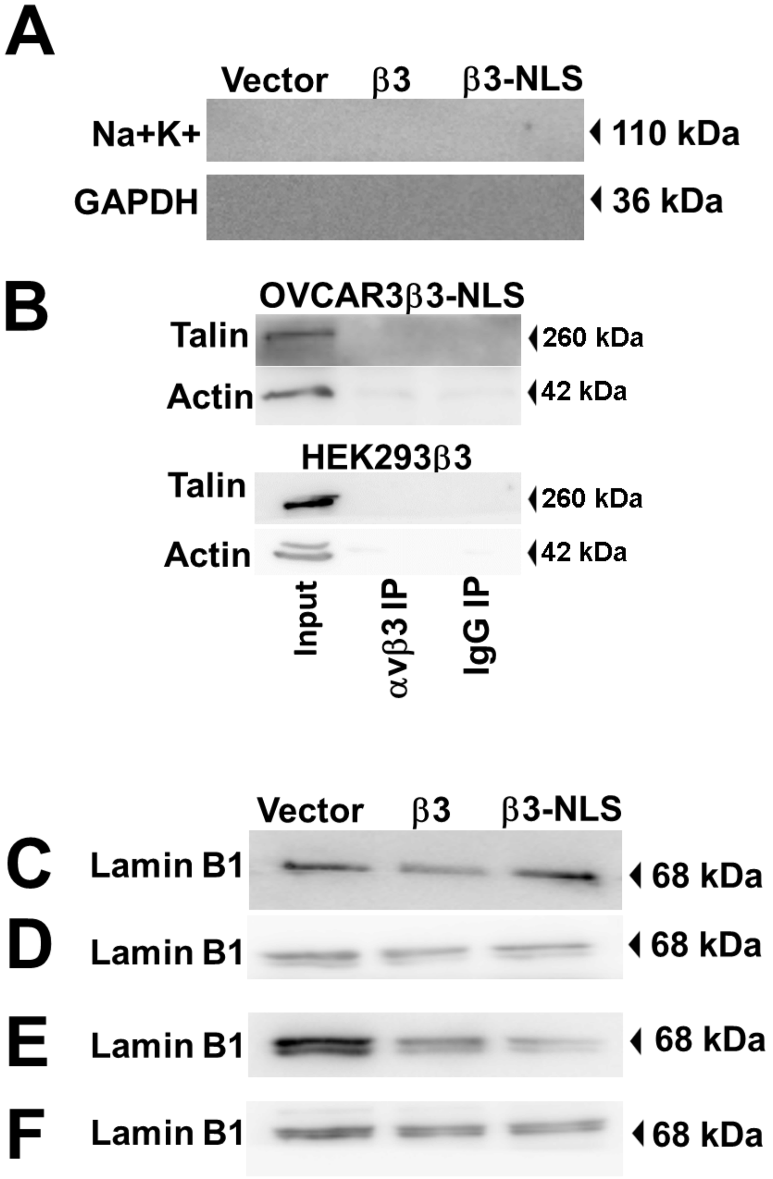

Supplementary Figure 4: Western blot analysis of nuclear fractions extracted from OVCAR3 cells transfected with an empty vector, β3 integrin and NLS-modified β3 integrin. Western blots analysis for **(A)** Nuclear fraction purity using Na+K+ and GAPDH controls. **(B)** Representative Western blots from OVCAR3 cells transfected with β3-NLS vector and HEK293β3 cells transfected with the native β3 following IP from nuclear and non-nuclear fractions using anti αvβ3 dimer or IgG control antibodies. Protein input and pull down fractions are depicted for actin and talin in the nuclear and non-nuclear fractions **(C-F)** Lamin B1 loading controls.

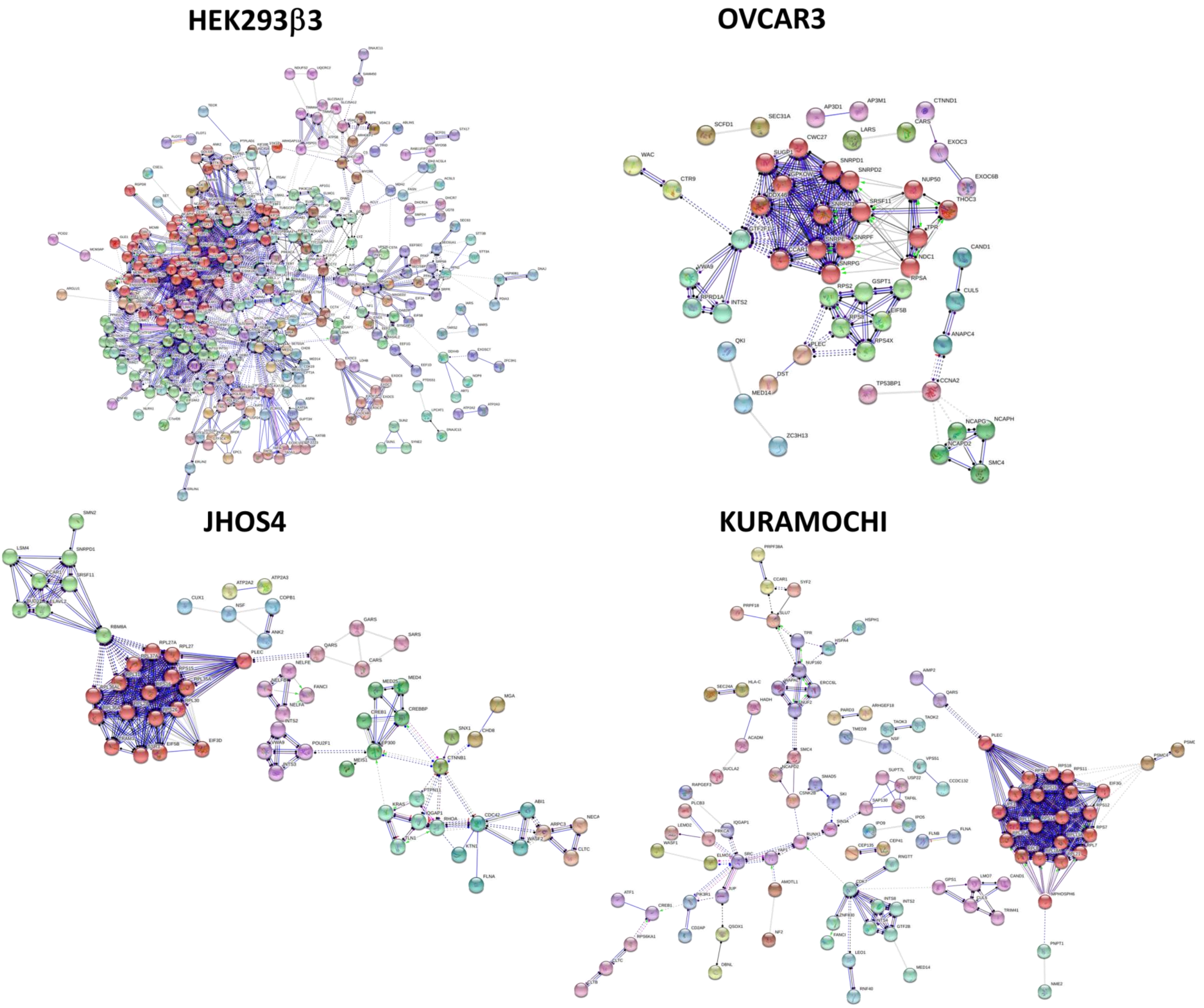

Supplementary Figure 5: Interaction network between the proteins which complexed with the nuclear αvβ3 in the various cell models. The online STRING tool was used to generate images (https://string-db.org).
